# Enhanced Healing of Diabetic Wounds by Subcutaneous Administration of Human Umbilical Cord Derived Stem Cells and Their Conditioned Media

**DOI:** 10.1155/2013/592454

**Published:** 2013-09-08

**Authors:** Chandrama Shrestha, Liling Zhao, Ke Chen, Honghui He, Zhaohui Mo

**Affiliations:** Department of Endocrinology and Metabolism, The Third Xiangya Hospital, Central South University, Changsha 410013, China

## Abstract

*Objective*. Mesenchymal stem cells (MSCs) isolated from the umbilical cord and their conditioned media (CM) can be easily obtained and refined compared with stem cells from other sources. Here, we explore the possibility of the benefits of these cells in healing diabetic wounds. *Methodology and Results*. Delayed wound healing animal models were established by making a standard wound on the dorsum of eighteen db/db mice, which were divided into three groups with six mice in each: groups I, II, and III received PBS, UC-MSC, and CM, respectively. UC-MSC and their CM significantly accelerated wound closure compared to PBS-treated wounds, and it was most rapid in CM-injected wounds. In day-14 wounds, significant difference in capillary densities among the three groups was noted (*n* = 6; *P* < 0.05), and higher levels of VEGF, PDGF, and KGF expression in the CM- and UC-MSC-injected wounds compared to the PBS-treated wounds were seen. The expression levels of PDGF-**β** and KGF were higher in CM-treated wounds than those in UC-MSC-treated wounds. *Conclusion*. Both the transplantation of UC-MSC and their CM are beneficial to diabetic wound healing, and CM has been shown to be therapeutically better than UC-MSC, at least in the context of diabetic wound healing.

## 1. Introduction 

Diabetes has undoubtedly become a major public health concern of the twenty-first century. Various studies have estimated the impact of diabetes, and it seems that the numbers are growing at unprecedented rates. The International Diabetes Federation claims that 366 million people had diabetes in 2011 and by 2030 this number will have increased to 552 million, and that this caused 4.6 million deaths in 2011 [1]. About 15% of people with diabetes suffer from foot ulcerations [2]. Diabetic wounds that resist healing are also associated with decreased peripheral blood flow and often resist current therapies. These achieve only 50% healing rates even with the best treatment available, that too, for a short-term [2], and 4/5 of these cases eventually succumb to amputation of the lower extremity [3, 4]. Normal wounds, without underlying pathological defects, heal readily, but the healing deficiency of diabetic wounds can be attributed to a number of factors, including decreased production of growth factors and reduced revascularization. Mesenchymal stem cells are multipotent, nonhematopoeitic progenitor cells that hold great promise for tissue regeneration. Mesenchymal stem cells isolated from the umbilical cord and their conditioned media can be easily obtained and refined compared to stem cells from other sources. Although the therapeutic potential of transplanted human umbilical cord derived stem cells has been widely explored as a promising tool in the treatment of several human diseases, including graft versus host disease [5–8], diabetes [9, 10], Crohn's disease [8], heart disease [11–13], and solid tumor cancers [14, 15], its effect on healing diabetic wounds has not been studied to the same degree. 

## 2. Materials and Methods 

### 2.1. Materials

#### 2.1.1. Cell Culture

*Isolation and Culture of Umbilical Cord Derived Mesenchymal Stem Cells*. Umbilical cords of gestational ages (39-40 weeks) were obtained from the Department of Obstetrics and Gynecology of The Third Xiangya Hospital of Central South University after normal deliveries. Tissue collection for research was approved by the institutional review board of The Third Xiangya Hospital of Central South University. After having been minced into 1-2 mm^3^ fragments, umbilical cord was incubated with 0.075% collagenase type II for 30 minutes and then 0.125% trypsin for 30 minutes with gentle agitation at 37 degrees centigrade. The digested mixture was then passed through a 100 micrometre filter to obtain cell suspensions. Cells were plated at a density of 1 × 10^6^ cells/cm^2^ in noncoated cell culture flasks. Growth medium consisted of Dulbecco's modified Eagle medium with low glucose and 20% fetal bovine serum supplemented with 4 ng/mL bFGF and 2 mM L-glutamine. Cultures were maintained in a humidified atmosphere with 5% carbon dioxide at 37 degrees centigrade. After 3 days of culture, the medium was replaced, and nonadherent cells were removed. The medium was then changed twice weekly then after. Once 80% of confluence had been reached, adherent cells were replated at a density of 1 × 10^4^/cm^2^ in umbilical cord growth medium for expansion. The primary culturing of UC-MSC in Dulbecco's modified eagle Medium until near confluence (passage 1) was approximately one week. The time course of UC-MSC was amplified for two weeks until the third passage, and UC-MSC were then applied to the wound bed.

*Immunophenotype Analysis*. The morphology of cells derived from umbilical cord was fibroblast-like as observed under the microscope. Surface markers including CD29, CD44, CD73, CD90, and CD105 were positive. CD34, CD45, CD31, and HLA-DR were negative. Cells were stained in a single label and then analyzed by flow cytometry with a fluorescent-activated cell sorter (FACS). 

*Preparation of Conditioned Media*. Conditioned media was derived from culturing of UC-MSCs in serum-free M199 media at 37°C for 24 hours and was cleared out and concentrated by centrifugation. After 24 hours, the supernatant was collected as conditioned media and filtered through a 0.2 *μ*m filter for immediate use. 

*Mesenchymal Stem Cells: Characterization and Their Differentiation Activity*. After mesenchymal stem cells were tested for their characterization, they were tested for their ability to differentiate into different mesenchymal lineages including adipocytes, osteoblasts, and chondrocytes. Adipogenic differentiation was induced with 1 × 10^−8^ mM dexamethasone and 5 microgram/mL insulin, and droplet staining was performed using oil red O. Osteogenic differentiation was induced by treating mesenchymal stem cells with 10^−8^ M dexamethasone, 10 mM beta-glycerol-phosphate, and 50 microgram/mL ascorbic acid, and differentiated cells were identified by Alizarin red staining. Chondrogenic differentiation medium was composed of high-glucose Dulbecco's modified Eagle medium supplemented with 40 microgram/mL proline, 50 mg/mL ITS-plus, 100 microgram/mL sodium pyruvate, GlutaMAX, 50 microgram/mL ascorbate-2-phosphate, 10 ng/mL transforming growth factor-beta 3, and 1 × 10^−8^ mM dexamethasone. Chondrogenic differentiation was visualized by Alcian blue staining. 

*Animal Model for Diabetic Wound Healing*. Eighteen male *db/db* mice (BKS.Cg-*m* +/+ *Lepr* ^*db*^/*J*, *db* ^+^ */db* ^+^; 10–14 weeks old; male; body weight, (42.4 ± 2.1) g; blood glucose > 16.67 mM) were obtained from Model Animal Research Center of Nanjing University. The animals were randomly divided into three groups (*n* = 6 per group), and the excisional wound-healing model was generated. The glucose levels were measured every alternate day during this experimental procedure in wound healing. The glucose level remained more than 16.67 mM in all mice in all groups, and there was no significant difference in the blood glucose levels among the three groups throughout the entire 14-day period of the experiment. The mice were sedated by intraperitoneal administration of 6% chloral hydrate (4 mL/kg body weight) before the procedure. Then the mice were shaved on the dorsum, and a full-thickness dorsal skin defect was created on the dorsal midline using a 6 mm diameter biopsy punch for the evaluation of wound healing. All the animals were treated humanely according to the guidelines provided in the Guide for the Care and Use of Laboratory Animals, published by the National Institutes of Health. All animals were housed individually under standard conditions. The study was approved by the Institutional Animal Care and Use committee of The Third Xiangya Hospital of Central South University. 

*Criteria for Inclusion*. Animals with random blood glucose which exceeded 16.67 mM were included in the study. The measurements were taken in triplicate.

### 2.2. Methods

#### 2.2.1. Cell Administration at the Wound Site

Right after wound induction, each wound received 2.0 × 10^6^ cells in 60 *μ*L of PBS injected subcutaneously along the margin of the dorsal wound at four injection sites applied onto the wound bed (*n* = 6). Conditioned medium was administered immediately and on every alternate day (*n* = 6), whereas an equivalent volume of PBS was administered in the control group (*n* = 6) in the same fashion. A transparent bioocclusive adhesive tape (Comfeel Plus Transparent Dressing) was placed over the wounds. The adhesive tape on the skin in mice was tested prior to this experiment for any skin irritation or allergic reaction, and there was none. The transparent dressing was changed every alternate day to maintain wet wound conditions. Wounded animals were housed individually under standard conditions. Wound healing was assessed by measuring the epithelial gap every alternate day for two weeks. A ruler was placed next to the wound, and the wounds were photographed from an equidistant arbitrary level at all times. Then the area was calculated using Image Pro Plus. Scale bar was taken as 1 mm. 

#### 2.2.2. Estimation of Wound-Healing Area

The wound-healing area was assessed once every alternate day after the procedure. At different time points (0, 2, 4, 6, 8, 10, 12, and 14 days) after wounding, lesion closure was documented using a digital camera. Images were processed and analyzed by tracing the wound margin and calculating the pixel area using the Image Pro Plus. Reepithelialization was reported as a percentage of the initial wound area and calculated as reepithelialization percentage = [1 − (area  *on*⁡  day  of  analysis/area  *on*⁡  day  0)] × 100. The day in which the full-thickness wound is seen to be completely closed was taken as the day of complete healing. The healed area was calculated from the original wound area (diameter = 6 mm) and the unhealed area once every alternate day for two weeks.

#### 2.2.3. Histological Examination

A full-thickness 3 mm punch biopsy was performed from the wound margin after the wounds healed completely, and euthanized. Six samples were randomly examined and analyzed in each group. Specimens were fixed in 10% formalin and embedded in paraffin. Sections were cut from the paraffin-embedded specimens and stained with hematoxylin and eosin. Images of hematoxylin and eosin stained slides of each wound obtained from maximal cross sections were digitally acquired, and then wound scoring was done (Table 1).

#### 2.2.4. RT-PCR Analysis

Total RNA (1 *μ*g) was extracted from wound tissues harvested at day 14 after wound induction and was processed for cDNA synthesis using the Superscript first-strand synthesis system (Invitrogen), after which the cDNA was amplified with 40 cycles of PCR using gene-specific primers as shown in Table 2. Real-time PCR was performed using Transtart Green qPCR SuperMix UDG system. Data analysis was based on the ΔΔCt method with normalization of the raw data to housekeeping gene, GAPDH, included in the experiment. All reactions were performed in triplicate.

#### 2.2.5. Immunohistochemistry

Wound sections were treated with 0.3% hydrogen peroxide to quench the endogenous peroxidase, and then antigen epitopes were retrieved by heating in Target Retrieval Solution. After sections were blocked in 10% normal goat serum, they were treated with anti-von Willebrand factor (vWF; Abcam, USA).

#### 2.2.6. Data Management and Statistical Analysis

Data were analyzed by SPSS 16.0 software and are presented as mean ± SEM. Student's paired *t*-test was performed for data comparison of paired samples, and analysis of variance followed by Bonferroni's post hoc multiple comparison test was performed to determine the significant differences among the three groups. A probability (*P*) value of less than 0.05 was considered statistically significant. 

## 3. Results

### 3.1. The Transplantation of UC-MSCs and Their CM Accelerate Wound Closure in Diabetic Mice

**Figure 1:**
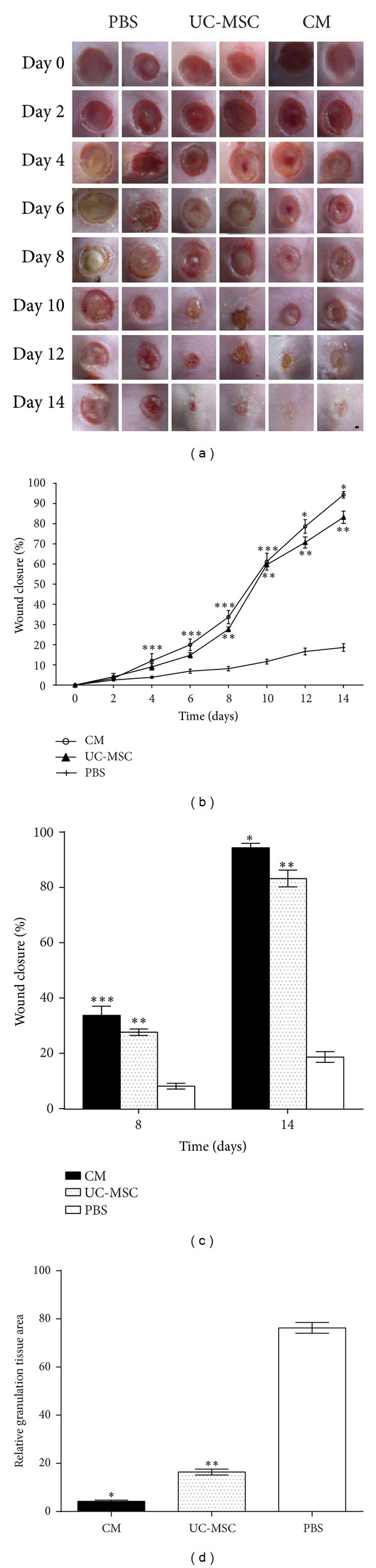
Effects of CM, UC-MSC, and PBS on wound closure. (a) Representative photographs of the wounds of every alternate day from day 0 to day 14 after injury. Scale bar is 1 mm. (b) Comparison of wound closure rates expressed as a percentage of its initial wound area every alternate day after wounding (*n* = 6; *P < 0.05, CM versus UC-MSC or PBS); (*n* = 6; **P < 0.05, UC-MSC versus PBS); (*n* = 6; ***P < 0.05, CM versus PBS). (c) Wound measurement of all groups at day 8 and day 14 (*n* = 6; *P < 0.05, CM versus UC-MSC or PBS); (*n* = 6; **P < 0.05, UC-MSC versus PBS). (d) Relative granulation tissue area of wounds treated with CM, UC-MSC, and PBS at day 14 (*n* = 6; *P < 0.05, CM versus UC-MSC or PBS); (*n* = 6; **P < 0.05, UC-MSC versus PBS).

The therapeutic effect of transplantation of umbilical cord derived mesenchymal stem cells and their conditioned media to heal wounds in genetically diabetic *db/db* mice was significant (Table 3). *db/db* mice in which PBS was injected displayed markedly delayed wound healing. When UC-MSCs and their CM were injected subcutaneously around full-thickness dermal wounds created on the diabetic mice, wound closure was significantly accelerated as early as day four after injury in the CM-treated wounds and at day eight after injury in the UC-MSC-treated wounds compared to PBS-treated ones and became more evident at day 14 (Figures 1(a), 1(b), and 1(c)). This significant increase in the healed wound area was consistently observed until day 14 (CM (94.38 ± 0.80)%, UC-MSC (70.71 ± 1.39)%, PBS (18.63 ± 1.13)% on day 14). Statistically significant difference in the wound-healing rate among all groups was observed on day 12 and day 14 (*P* < 0.05). At day 14, all 6 wounds in CM-treated *db/db* mice and 3 of 6 wounds in UC-MSC-treated wounds achieved complete closure, but no completely closed wound was seen in PBS-treated mice (*n* = 6). In addition, substantially reduced cross-sectional area of granulation tissue among all groups was observed at day 14 (*P* < 0.05) (Figure 1(d)).

**Figure 2:**
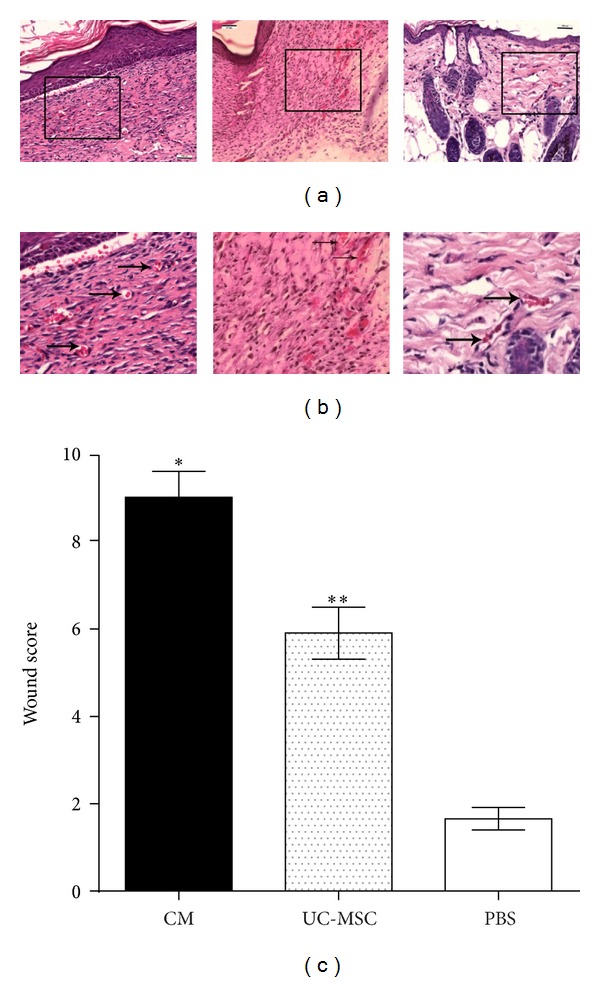
Histological analysis of day-14 wounds in *db/db* mice. Scale bar is 100 *μ*m. (a) H and E stained images of CM-, UC-MSC- and PBS-injected tissues from left to right, respectively (20x). (b) Magnified images of CM-, UC-MSC-, and PBS-injected tissues from left to right, respectively. (c) Wound scores at day 14 (*n* = 6; *P < 0.05, CM versus UC-MSC or PBS); (*n* = 6; **P < 0.05, UC-MSC versus PBS).

Histological evaluation of wounds in *db/db* mice at 14 days disclosed enhanced re-epithelialization in conditioned media treated wounds (complete epithelialization in all 6 wounds examined; *n* = 6) compared with UC-MSC-treated (complete reepithelialization in 3 of 6 wounds; *n* = 6) or PBS-treated wounds (complete reepithelialization in none; *n* = 6). Analysis of wounds on day 14 indicated wounds treated with conditioned media and UC-MSC had increased vasculature compared to PBS-treated controls (Figure 2(a)). In addition, granulation tissue in conditioned media and UC-MSC-treated wounds appeared to be thicker but lesser in area. Consistent with these findings, the wound scores of the day-14 wounds among three groups were statistically significant (Figure 2(b)). 

### 3.2. Injection of UC-MSC and CM Increases Neovascularization of Wounded Tissue

**Figure 3:**
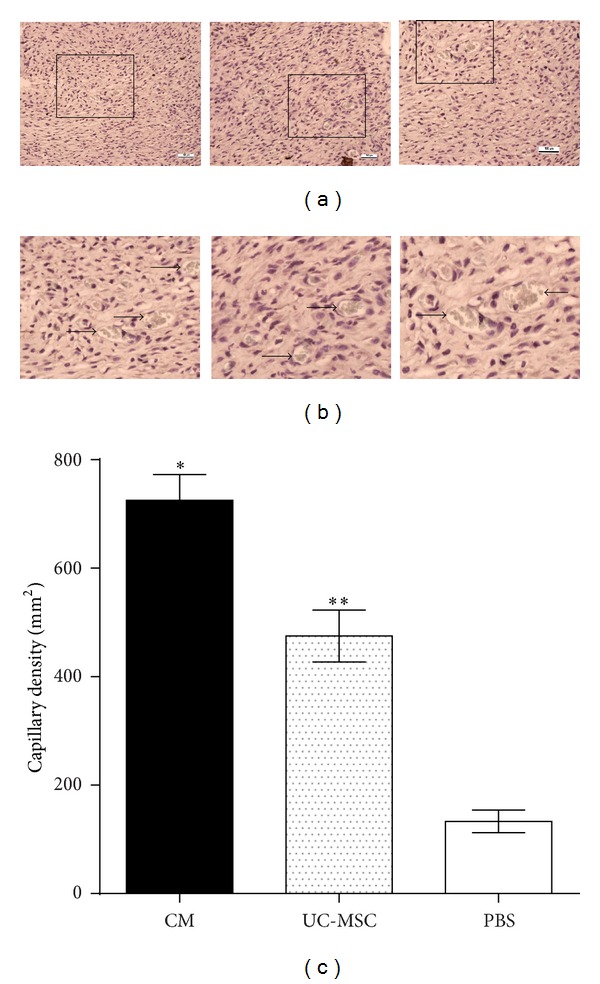
Capillary density in 14-day-old wounds was determined after immunohistochemistry. Scale bar is 100 *μ*m. (a) Images of CM-, UC-MSC-, and PBS-injected tissues from left to right, respectively (20x). (b) Magnified images of CM-, UC-MSC-, and PBS-injected tissues from left to right, respectively. (c) Capillary density as the number of vWF-positive vessels per mm^2^ (*n* = 6; *P < 0.05, CM versus UC-MSC or PBS); (*n* = 6; **P < 0.05, UC-MSC versus PBS).

Capillary densities in day-14 wounds were assessed after immunohistochemical staining for vWF. Immunohistological staining of tissue sections for vWF showed increased vasculature in conditioned media treated wounds at 14 days compared with UC-MSC-treated or PBS-treated wounds (Figures 3(a) and 3(b)). Significantly higher capillary density in conditioned media treated (725 ± 47.87/mm^2^) and in UC-MSC-treated (475 ± 47.87/mm^2^) wounds compared to PBS-treated (133.3 ± 21.02/mm^2^) wounds was found (*n* = 6; *P < 0.05). 

### 3.3. Secreted Factors from Mesenchymal Stem Cells and CM Directly Stimulate Growth Factors That Are Potentially Relevant to Dermal Healing

**Figure 4:**
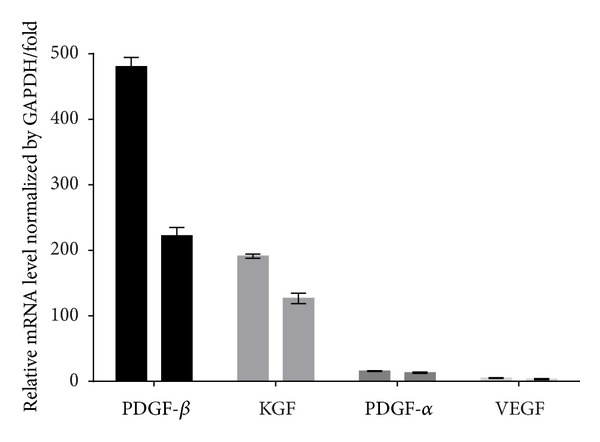
The mRNA profile of wound-healing related growth factors analyzed by real-time PCR and normalized by its relative ratio to GADPH in CM-treated (left) and UC-MSC-treated (right) wounds.

To determine whether mesenchymal stem cells and conditioned media derived from culturing of UC-MSCs in serum-free M199 media for 24 hours could enhance angiogenesis through a paracrine effect, real-time polymerase chain reaction analysis was performed on wound tissues harvested at 14 days and normalized as its relative ratio to GAPDH. It revealed higher levels of VEGF, PDGF, and KGF expression in the CM- and UC-MSC-injected wounds compared to the PBS-treated wounds. The expression of PDGF-*β* and KGF was higher in CM-treated wounds compared to UC-MSC-treated wounds. The data were obtained with samples from three independent preparations and are expressed as mean ± SEM (Figure 4).

## 4. Discussion

There is certainly no room for doubt that innovative treatments for the prevention, alleviation, and/or total cure of the diabetic wounds are in high demand. Here, we show that umbilical cord derived mesenchymal stem cells enhance wound healing in diabetic mice by promoting reepithelialization, secretion of paracrine factors, and neovascularization. 

Isogenic strains of mice were used in the experiment. These are considered immortal clones of genetically identical animals. The purity of the mouse stock can assure a research scientist of a true and sure experiment, and in experimental medicine today, the use of inbred genetic materials is just as necessary as the use of the aseptic and antiseptic precautions in surgery [16]. Gruneberg in 1952 further emphasized the use of inbred strains saying that the introduction of inbred strains into biology is probably comparable in importance with that of the analytical balance into chemistry [17]. We used an excisional wound-healing model in genetically diabetic *db/db* mice, which has been known to have markedly impaired wound healing and is an established model to study the effect of therapeutic reagents on wound healing [18–22]. *db/db* mouse model is characterized by early hyperinsulinemia with marked hyperglycemia progressing with age to slowly developing islet failure. These animals exhibit hyperglycemia over 16.67 mM by six weeks of age which increases in severity over time. Our study shows that wound closure is significantly delayed in diabetic mice, and umbilical cord derived mesenchymal stem cell transplantation significantly accelerates wound healing in diabetic mice in this study. Consistent to our findings, similar studies, albeit with different sources of mesenchymal stem cells, including those from the bone marrow [18, 21], umbilical cord blood [23], and adipose tissue [24], also accelerated wound healing in diabetic mice. Other studies which reiterate the efficacy of these autologous stem cells in wound healing in patients have also been reported [25–30]. 

Allogeneic mesenchymal cells have been administered by various routes in animal wound-healing models [31–36]; however, the optimal type has not been well defined. These include topical [37, 38], intravenous [36], local injection [18, 31, 34, 36], and systemic administration [27, 37]. In most studies, the cells were delivered in a single dose; however, one study reported a study with multiple doses [28]. No significant adverse effect has been reported so far in case of allogeneic lineage negative bone marrow cells, despite being effective in wound healing in a murine diabetic wound-healing model [38]. In addition, in consistency with to our findings, human adipose tissue derived stem cells delivered to diabetic (*db/db*) mice have shown effectiveness in wound healing, also without adverse effect [39]. In studies by Badiavas et al., mixed population of bone marrow cells were used to treat chronic wound patients via local injection and topical application in saline, and they state that no adverse events were noted [40, 41]. Variation in dosing in these studies range from single to multiple applications with doses up to 2 × 10^8^ cells per administration. Falanga et al. came up with the finding that dosages exceeding 1 × 10^6^ cells·cm^−2^ were directly related to accelerated wound closure [28]. 

The immunomodulatory properties of mesenchymal stem cells allow for an allogeneic source for therapy. Possible drawbacks of autologous source of the same can be reflected in chronic disorders associated with nonhealing wounds such as diabetes and autoimmune disease, owing to the abnormalities in bone marrow cells, including mesenchymal stem cells [42–46]. Bone marrow derived cells from chronic wound patients showed reduced growth in culture compared to their normal counterparts [41], and the administration of allogeneic MSCs could be preferred under such circumstances [44–47]. The therapeutic benefits from healthy donors of allogeneic mesenchymal stem cells might surpass those that can be accomplished by the transplantation of autologous mesenchymal stem cells derived from chronic wound patients.

Neovascularization, the formation of new blood vessels which is necessary to sustain the newly formed granulation tissue and the survival of keratinocytes, is considered as one of the important processes in wound healing [3, 48, 49]. In this study, we demonstrated that MSC-treated wounds had enhanced capillary density, suggesting that these cells promote angiogenesis. It was also found that UC-MSC and CM injection resulted in increased amounts of KGF and PDGF in the wounds. The levels of PDGF-*β* and KGF were even more pronounced in CM-injected wounds than UC-MSC-injected wounds in this study. Indeed, another important role in angiogenesis is played by vasculoendothelial growth factor, which does so by stimulating endothelial cell proliferation, migration, and organization into tubules [49, 50]. Moreover, VEGF increases circulating endothelial progenitor cells [50]. VEGF was comparatively more in UC-MSC- and CM-injected wounds than in PBS-treated wounds in this study too. Angiogenesis was more pronounced in CM-injected wounds compared to UC-MSC, but the levels of VEGF were found to be similar in both groups. VEGF levels were measured in day-14 wounds only. One possible reason could be that CM was administered every alternate day, and it enabled stable and effective long-term release of VEGF compared to UC-MSC and, as a consequence, more pronounced angiogenesis compared to UC-MSC.

Angiogenesis is a complex process controlled by the balance of proangiogenic and antiangiogenic factors [50]. Our results suggest that UC-MSCs engrafted in the wound release proangiogenic factors, subsequently leading to MSC-mediated enhanced angiogenesis. Differentiation of MSCs into keratinocytes found in our study was consistent with the findings of similar other studies [51, 52]. In our study, we found that injection of CM could accelerate wound closure, and the enhancement was even better and more rapid than that achieved by UC-MSC transplantation in contrast to the results obtained by Wu et al. [18], although they used MSC from a different source. These results suggest that differentiation of MSCs may be one of the major role players in MSC-mediated cutaneous repair/regeneration, although paracrine factors are important. 

Several limitations do exist in our study. Human MSCs are likely to play unique roles in delivery to nonhealing wounds that cannot be fully duplicated in animal models. Animal models of chronic wounds are just delayed-healing models, with marked differences in pathophysiology [53–55]. Wounds of size surpassing 5 cm^2^ in humans and lasting for a duration exceeding six months [56, 57] and age-related changes and chronic disorders common in chronic wound patients are next to impossible to be studied in animals [58]. Elderly patients with chronic wounds are more likely to respond to allogeneic therapy owing to the immune dysregulation in such patients [59]. These important factors of mesenchymal stem cells, compounded with the imperfections inherent to animal model of chronic wounds, show the importance of more extensive investigation in humans. 

Controversial data concerning the effects of MSCs on regulation of tumor growth have been reported for animal and *in vitro* models [60–63]. The tumorigenicity of transplanted cells needs thorough assessment, although no tumor formation has been reported so far, after UC-MSCs transplantation [64].

Our study demonstrates the beneficial effect of UC-MSCs and CM in cutaneous regeneration and wound healing in diabetic mice through angiogenesis and paracrine effects. UC-MSCs and CM represent a defined and expandable population of cells with potential therapeutic use in the treatment of diabetic wounds, and CM has proved to be therapeutically better, at least, in the context of diabetic wound healing in this study.

## 5. Conclusion

Both the transplantation of UC-MSC and CM accelerate wound closure in diabetic mice, and even more rapid rate of wound healing is achieved in CM-treated wounds than MSC-treated wounds. Secreted factors from UC-MSC and CM directly stimulate growth factors (VEGF, PDGF, and KGF) that are potentially relevant to dermal healing. PDGF-*β* and KGF levels were more pronounced in CM-injected wounds than in UC-MSC-injected wounds. Injection of UC-MSC and CM increases angiogenesis of wounded tissue. The angiogenesis in wounded tissue after CM administration was more than that achieved by UC-MSC administration.

## Figures and Tables

**Table 1:** Criteria for wound scoring.

Score	Epidermal and dermal regeneration	Granulation tissue	Angiogenesis (day-14 wounds only)
1–3	Minimal to moderate reepithelialization	Granulation around wound edges only	Capillary density <300/mm^2^
4–6	Complete reepithelialization	Granulation around wound edge and in 30%–50% of wound bed	Capillary density 300–500/mm^2^
7–9	Complete reepithelialization	Thick granulation around wound edge and in >50% of wound bed	Capillary density >500/mm^2^

http://www.plosone.org/article/info%3Adoi%2F10.1371%2Fjournal.pone.0041105#pone-0041105-t001, at doi: 10.1371/journal.pone.0041105.t001.

**Table 2:** Primers used in PCR.

Primer name	Primer sequence (5′-3′)
VEGF	F: CAAGGCCAGCACATAGGAGA
	R: AGGGAACGCTCCAGGACTTA
PDGF-*β*	F: TCGAGA TTGTGCGGAAGAAG
	R: GTGTGCTTGAATTTCCGGTG
PDGF-*α*	F: CCATTCGGAGGAAGAGAAGC
	R: GTATTCCACCTTGGCCACCT
KGF	F: TTCACATTATCTGTCTAGTGGGT
	R: TGGGTCCCTTTTACTTTGCC
GAPDH	F: GAAGGTGAAGGTCGGAGTC
	R: GAAGATGGTGATGGGATTTC

**Table 3:** Analysis of wound closure rate of all groups (mean ± SEM).

Duration (days)	Area CM (%)	Area UC-MSC (%)	Area PBS (%)
0	0	0	0
2	3.31 ± 0.67	4.13 ± 0.81	2.51 ± 0.09
4	12.01 ± 1.77***	9.10 ± 0.82	3.84 ± 0.32
6	19.98 ± 1.41***	14.75 ± 0.66	6.9 ± 0.60
8	33.72 ± 1.67***	27.63 ± 0.59**	8.11 ± 0.61
10	61.20 ± 2.12***	59.70 ± 0.41**	11.64 ± 0.68
12	78.62 ± 1.69*	70.71 ± 1.39**	16.67 ± 0.96
14	94.38 ± 0.80*	83.22 ± 1.54**	18.63 ± 1.13

*Note*. Comparision of wound closure rates (*n* = 6;  *P < 0.05, CM versus UC-MSC or PBS); (*n* = 6;  **P < 0.05, UC-MSC versus PBS); (*n* = 6;  ***P < 0.05, CM versus PBS).

## Abbreviations

CM: Conditioned media
GAPDH: Glyceraldehyde-3-phosphate dehydrogenase
KGF: Keratinocyte growth factor
MSCs: Mesenchymal stem cells
PBS: Phosphate buffer solution
PCR: Polymerase chain reaction
PDGF-*α*: Platelet-derived growth factor-alpha
PDGF-*β*: Platelet-derived growth factor-beta
UC-MSCs: Umbilical cord derived mesenchymal stem cells
VEGF: Vascular endothelial growth factor
vWF: von Willebrand factor.
